# How valid are women’s reports of the antenatal health services they receive from Community Health Workers in Gombe State north-eastern Nigeria?

**DOI:** 10.1186/s12884-022-05220-x

**Published:** 2022-12-03

**Authors:** Emmanuel Olal, Nasir Umar, Jennifer Anyanti, Zelee Hill, Tanya Marchant

**Affiliations:** 1 Department of Disease Control, London School of Hygiene and Tropical Medicine, Keppel Street, London, WC1E 7HT UK; 2 Yotkom Uganda, Awich Road, Kitgum, Uganda; 3 Society for Family Health, Justice Ifeyinwa Nzeako House, 8 Port Harcourt Crescent, Area 11, Garki, Abuja, Nigeria; 4 Institute of Global Health, University College London, London, UK

**Keywords:** Community Health Workers, Counselling skills, Validity of women's responses

## Abstract

**Background:**

Community health workers (CHWs) in low- and middle-income countries are key to increasing coverage of maternal and newborn interventions through home visits to counsel families about healthy behaviours. Household surveys enable tracking the progress of CHW programmes but recent evidence questions the accuracy of maternal reports. We measured the validity of women’s responses about the content of care they received during CHW home visits and examined whether the accuracy of women’s responses was affected by CHW counselling skills.

**Methods:**

We conducted a criterion validity study in 2019, in Gombe State-Nigeria, and collected data from 362 pregnant women. During accompanied CHW home visits the content of CHW care and the presence or absence of 18 positive counselling skills were observed and documented by a researcher. In a follow-up interview three months later, the same women were asked about the care received during the CHW home visit. Women’s reports were compared with observation data and the sensitivity, specificity, and area under receiver curve (AUC) calculated. We performed a covariate validity analysis that adjusted for a counselling skill score to assess the variation in accuracy of women’s reports with CHW counselling skills.

**Results:**

Ten indicators were included in the validity analysis. Women consistently overestimated the content of care CHWs provided and no indicator met the condition for individual-level accuracy set at AUC ≥ 0.6. The CHW counselling skill score ranged from 9–18 points from a possible 18, with a mean of 14.3; checking on client history or concerns were the most frequently missed item. There was evidence that unmarried women and the relatively most poor women received less skilled counselling than other women (mean counselling scores of 13.2 and 13.7 respectively). There was no consistent evidence of an association between higher counselling skill scores and better accuracy of women's reports.

**Conclusions:**

The validity of women's responses about CHW care content was poor and consistently overestimated coverage. We discuss several challenges in applying criterion validity study methods to examine measures of community-based care and make only cautious interpretation of the findings that may be relevant to other researchers interested in developing similar studies.

**Supplementary Information:**

The online version contains supplementary material available at 10.1186/s12884-022-05220-x.

## Introduction

Community health workers (CHWs) are key for scaling-up access to and uptake of essential maternal and newborn health (MNH) interventions in low- and middle-income countries (LMICs) [1–3]. Timely and quality MNH interventions during pregnancy, birth and postnatal periods increase maternal and newborn survival [4–6] and CHW home visits are a widely adopted strategy to deliver health education messages and promote positive practices like health care utilisation amongst families. In some settings, CHWs also detect and treat illnesses and provide commodities such as mosquito nets and iron folate [7, 8].

It is common for the activities that are undertaken by CHWs during home visits to be described under the umbrella of counselling. While relatively little is known about the counselling skills that best support health education messaging in LMICs, there is evidence to suggest that sub-optimal counselling skills limit the effectiveness of home-visit interactions [9–12]. It is generally understood that counselling is made more effective through strong interpersonal communication skills, active listening and connecting with clients, to translate health information into the local context [8, 13, 14]. But programmatic evidence on counselling behaviours during CHW home visits is very limited.

Multiple data sources are needed to effectively assess CHW performance and community-level outcomes [15], including periodic household survey data such as the Demographic Health Surveys (DHS) and Multiple Indicator Cluster Surveys (MICS) [16–18] which provide useful information for health decision making [19, 20]. However recent studies have questioned the accuracy and reliability of maternal recall during such surveys, especially for pregnancy and childbirth events [19, 21, 22]. Women tend to recall abstract concepts like health education messages less frequently than preventive interventions [23]; a few studies have also shown that women find survey questions on antenatal or perinatal care confusing [24]; and relatively little is known about the validity of indicators on counselling for health education messages, for example, the validity of questions in both DHS and MICS asking: “during the first two days after birth, did any health care provider counsel you on danger signs for newborns; counsel you on breastfeeding?”.

Our study, therefore, aimed to measure the validity of women's responses to questions about the content of care that they received during CHW home visits in Gombe State, north-eastern Nigeria. In the context of our study, the content of care encompassed CHW health education messages plus carrying out the verbal or physical health checks that they were trained to do. Further, we examined the counselling skills that CHWs displayed during home visits and assessed whether these skills affected the accuracy of women’s responses about the content of care.

## Materials and methods

### Study setting

Between June and October 2019 this study was nested within a measurement, learning and evaluation project implemented in Gombe State, Nigeria [25], a setting that continues to experience very high maternal, newborn and child mortality [26]. In 2017 the State had launched a CHW scheme to strengthen healthcare-seeking and provision of basic maternal and newborn health services, described in detail elsewhere [27]. A brief explanation of the scheme follows.

### CHW scheme in Gombe State

In brief, under the leadership of the Gombe State Primary Health Care Development Agency, the CHW scheme (referred to in Gombe as the Village Health Worker Scheme) recruited laywomen aged 18–49 years, trained them on a range of key MNH services and counselling skills, and deployed them to work with families in their communities [27]. During door-to-door home visits, CHWs were expected to identify pregnant women from within their catchment area and then make at least four home visits during pregnancy plus two during the first few weeks after birth, and link women to their primary health facility. At these home visits, CHWs carried and referred to a printed manual that contained health education messages and illustrations about care during pregnancy, intrapartum and postnatal periods [27]. This printed manual contained a total of 60 health education messages and health checks (Additional file 1), but each home visit had a defined sub-set of activities for CHWs to undertake. For example, promoting facility-based antenatal care at the first pregnancy visit, checking danger signs and recommending facility delivery at subsequent pregnancy visits, and promoting breastfeeding after delivery.

### Study design

Our study applied the criterion validity method. First, observed home visits were carried out to generate a reference (gold) standard. Three months later, interviewers returned to the same pregnant women for a follow-up interview and applied a structured questionnaire to ask them about the content of care received during the observed CHW home visit.

### Sample size

For estimation of the study sample size, a 50% prevalence of coverage observations (reference standard) was set for all indicators because of expected variability in the frequency of indicators. Sensitivity and specificity were each set at 70% ± 7.5% precision, and type 1 error set at 0.05, assuming an approximation to a binomial distribution. This approximated a sample size of 400 paired observations.

### Selection of participants

For 400 paired observations, twenty [19] CHWs were selected, each visiting 20 pregnant women in their catchment area. CHWs were selected from the Gombe government list of CHWs through simple random sampling. All the CHWs in the study population were numbered in consecutive order from 1 to n, where n was the total number of CHWs. Afterwards, n was divided by the required sample size to give 20 CHWs. A random number was selected between 1 and the sampling interval to select the first CHW. Subsequent CHWs were selected from the line list by adding the sampling interval to the preceding CHW position to obtain the next CHW until all remaining 19 CHWs were chosen. CHW client records were then used to select 20 women participants for each of the CHWs. To avoid possible selection bias introduced by CHWs, the names and addresses of all currently pregnant women logged in CHW record books before June 2019 were documented until a list of 400 pregnant women had been generated.

### Data collection procedures

Data collection tools were written to hand-held digital devices using “Census Survey Processing System (CSPro)” software. Following a review of past CHW performance from monitoring books, it was anticipated that accompanying 20 CHWs for two weeks would result in approximately 400 home visit observations.

One independent observer was assigned to accompany each CHW on the home visits. The observers were health practitioners with a broad understanding of CHW activities who were recruited through a competitive and open recruitment process conducted by the research team. They were trained to achieve an interrater reliability threshold of 80% against a simulated reference standard before participation in the study.

Consistent with the protocol for CHW home visits, family members were invited to be present during the observed CHW visit. Three types of data were collected. The observers recorded the sociodemographic characteristics of the visited women for descriptive analysis and to generate a relative measure of socio-economic status using principal components analysis (Table 1). Observers recorded their assessment of the CHW counselling skill (Table 2) using a checklist derived from the World Health Organization handbook for building counselling skills, adapted to the local setting [28]. Also, observers completed a checklist that had been developed to reflect the 60 health education messages and health checks that CHWs had been trained to deliver (Additional file 1); this checklist was based on the CHW manual and it was expected that the content of care would be tailored to the stage of the woman being visited. The checklist was phrased to ask observers to document whether a health education message or health check took place. For example, the observation checklist asked observers to record: “during the home visit, did the CHW specifically provide a telephone number for the Emergency Transport Scheme?”.

**Table 1:** Characteristics of women observed during CHW home visits in Gombe State

Maternal Characteristics *N* = 362		n (%)
Age (years)	≤ 18 years	29 (8)
	19–24	145 (40)
	25–29	92 (25)
	30–34	60 (17)
	≥ 35 years	36 (10)
Gestation age at observed visit	< 4 months	40 (11)
	4–6 months	154 (43)
	> 6 months	168 (46)
Number of children at the time of observation	0	66 (18)
	1	64 (18)
	2	72 (20)
	3	58 (16)
	≥ 4	102 (28)
ANC attendance at least once this pregnancy	Yes	273 (75)
	No	89 (25)
Marital status	Currently married	342 (95)
	Not currently married	20 (6)
Attended formal school	Yes	180 (50)
	No	182 (50)
Relative wealth quintiles^a^	Q1 – most poor	73 (20)
	Q2	74 (21)
	Q3	68 (19)
	Q4	72 (20)
	Q5 – least poor	71 (20)

^a^Total (N) is 358 because 4 participants had missing household asset data

**Table 2:** Observed^a^ CHW counselling skills during home visits

Positive counselling skills during the home visit	*N* = 362 n (%)
Made an appropriate introduction of herself and other people present	356 (98.3)
Explained the purpose of the home visit	337 (93.1)
Attempted to enlist family support (e.g., welcomed family members to sit in)	147 (40.6)
Reviewed/checked client health record/history	119 (32.9)
Checked for any concerns or problems of importance to the woman today	168 (46.4)
Used language the woman was likely to understand	362 (100)
Used appropriate counselling aids to support messages	355 (98.1)
Was knowledgeable and confident about the health messages	354 (97.8)
Responded in an understanding way when the woman talked	338 (93.4)
Probed for additional information when in conversation with the family	148 (40.9
Gently challenged any beliefs that were contrary to health messages	209 (57.7)
**Non-verbal behaviours of CHWs during the home visit**	
Smiled and greeted the woman and family on arrival	361 (99.7)
Made eye contact during discussions	359 (99.2)
Spoke in a clear, audible voice	361 (99.7)
Seated herself appropriately, allowing face-to-face interactions	358 (98.9)
**Positive counselling skills to conclude the home visit**	
Checked understanding of key messages by asking for feedback	266 (72.9)
Summarised the content of the discussion by emphasising key points	207 (57.2)
Asked the woman or family if they had final concerns or to ask questions	148 (40.9)

^a^Observations were made of 20 CHWs by 20 trained observers during a total of 362 home visits

During the observed home visit, pregnant women were issued unique identification cards. Three months after the observed home visit, the research team made a follow-up visit to the same women. Different personnel from the original observers were deployed and they used the unique identification cards to confirm the identity of women. Women were interviewed in private, away from other family members or friends who may have been present during the original CHW visit. The follow-up questionnaire reflected all 60 items on the observation checklist but questions to women were phrased to directly refer to the accompanied CHW visit three months before. For example, women were asked, “During the home visit by the CHW when my colleague was also present, did the CHW specifically provide you with a telephone number for the Emergency Transport Scheme?”.

### Data analysis

#### Matching records

Data were analysed using STATA/SE 16.1 statistical package. Records in the observation dataset were matched to records in the follow-up interview dataset using the unique identification numbers allocated to each participant and cross-checked: none-matched records were documented and dropped from further analysis.

#### Generating a score of the observed CHW counselling skills

The 18 variables on the observation of positive counselling skills were tabulated (Table 2). To summarise these data, an unweighted score was generated: all variables were coded as 1 for a positive response and 0 for a negative response then summed to generate a common unweighted counselling score with values 0–18. ANOVA tests were performed to examine the mean counselling score variation with selected maternal characteristics: age, parity, marital status, facility ANC visits in this pregnancy, formal education, and relative wealth quintiles (Table 3).

**Table 3:** Variation in CHW counselling score by women’s characteristics

Characteristic		Mean counselling score	Number of women *N* = 362	ANOVA *p*-value
Age (Years)	< 18	14.1	29	0.098
	19–24	14.1	145	
	25–28	14.9	92	
	29–34	14.5	60	
	> 35	13.7	36	
Gestation age at observed visit	< 4 months	14.4	40	0.627
	4–6 months	14.2	154	
	> 6 months	14.4	168	
Number of children at the time of observation	0	14.0	66	0.475
	1	14.1	64	
	2	14.2	72	
	3	14.7	58	
	≥ 4	14.5	102	
ANC attendance at least once this pregnancy	Yes	14.6	273	< 0.001
	No	13.4	89	
Marital status	Married	14.4	342	0.037
	Not currently married	13.2	20	
Attended formal school	Yes	14.2	180	0.246
	No	14.5	182	
Relative wealth quintiles^a^	Q1 – most poor	13.7	72	0.042
	Q2	14.3	72	
	Q3	14.0	71	
	Q4	14.6	73	
	Q5 – least poor	14.9	70	

^a^Total (N) is 358 women visited by *N* = 20 CHW; 4 participants had missing household data

### Validity analysis

For each of the 60 CHW content of care indicators, the following steps were taken. Frequencies were tabulated for the observed reference standard and women’s responses separately. The number of don't know responses was examined then dropped from further analysis. Two by two (2 × 2) contingency tables were constructed by cross-tabulation of the reference standard measures and the woman's self-reports. Agreement between the reference standard and self-reported measures was tabulated by calculating the number of responses with yes/yes and no/no agreement (see results for all indicators in the Additional file 1). For the validity analysis, only indicators with greater than 5 values per cell in the 2 × 2 tables were analyzed to avoid the inclusion of sparse data that could lead to inaccurate estimation [29]. Sensitivity, specificity, the area under receiver curve (AUC) were calculated (Table 4). Consistent with previous criterion validation studies, any AUC value below 0.6 was assessed to be as good as guesswork.

**Table 4:** Validity analysis of maternal reports regarding CHW content of care during home visits in Gombe State, Nigeria

**Indicator**	**Observed coverage** **N (%)**	**Women’s report** **N (%)**	**Matched Pairs** **N**	**Don’t Know** **N**	**Agreement %** **(95% CI)**	** ≥ 5 counts per cell**	**Sensitivity %** **(95% CI)**	**Specificity %** **(95% CI)**	**AUC** **(95% CI)**	**ROC regression coefficient**^a^ **(β 95% CI)**
	*N* = 362									
**CHW health education messages about birth preparedness and facility delivery**										
Provided an emergency telephone number	173 (47.7)	258 (72.4)	356	6	50.0 (44.7–55.3)	Y	73.3 (66.0–79.7)	28.3 (21.9–35.4)	0.51 (0.46–0.55)	0.006 (-0.019–0.031)
Advised to prepare skin disinfectant fluid	103 (28.4)	338 (93.8)	360	2	30.3 (25.5–35.1)	Y	92.2 (85.3–96.6)	5.4 (3.0–9.0)	0.49 (0.46–0.52)	-0.003 (-0.014–0.008)
**CHW health education messages about newborn danger signs and illness**										
Pneumonia is a serious illness of the lungs	296 (81.7)	345 (96.6)	357	5	82.4 (78.3–86.5)	Y	97.6 (95.2–99.0)	11.1 (4.6–21.6)	0.54 (0.50–0.58)	-0.002 (-0.035–0.032)
The Baby's cord is red is a danger sign	280 (77.3)	349 (96.6)	361	1	77.0 (72.6–81.4)	Y	97.5 (94.9–99.0)	6.2 (2.0–13.8)	0.52 (0.49–0.55)	0.02 (-0.003–0.044)
**CHW health education messages to pregnant women about other maternal illnesses**										
HIV infection	296 (81.7)	343 (95.0)	361	1	82.3 (78.6–86.5)	Y	97.3 (94.7–98.8)	15.4 (7.6–26.5)	0.56 (0.52–0.61)	-0.019 (-0.058–0.02)
Hypertension	296 (81.7)	345 (95.3)	362	0	80.4 (76.3–84.5)	Y	96.3 (93.4–98.1)	9.1 (3.4 -18.7)	0.53 (0.49–0.56)	0.014 (-0.018–0.047)
Diabetes	268 (74.0)	335 (93.8)	357	5	75.3 (70.8–79.9)	Y	96.3 (93.2–98.2)	13.3 (7.1–22.1)	0.55 (0.51–0.59)	0.02 (-0.011–0.052)
**CHW health checks or therapeutic care carried out during the home visit**										
Measured woman’s blood pressure	81 (22.3)	283 (78.3)	361	1	37.9 (32.9–42.9)	Y	86.4 (77.0–93.0)	23.9 (19.1–29.4)	0.55 (0.51–0.60)	0.021 (0.001–0.041)
Dispensed chlorhexidine	30 (8.2)	225 (62.3)	361	1	41.4 (36.3–46.5)	Y	73.3 (54.1–87.7)	38.7 (33.4–44.2)	0.56 (0.48–0.64)	-0.011 (-0.032–0.009)
Dispensed misoprostol	25 (8.0)	224 (61.8)	362	0	42.2 (36.1–46.3)	Y	72.0 (50.6–87.9)	38.9 (33.6–44.3)	0.55 (0.46–0.65)	-0.016 (-0.037–0.004)

^a^ROC regression coefficient for the relationship with CHW counselling skill score

### Covariate analysis

The observed CHW counselling skills were hypothesized to influence the accuracy of maternal reports on the content of CHW care at the follow-up interview. To examine this, we performed a covariate validity analysis that adjusted for the counselling score using the rocreg function in Stata 16.1E.

## Results

### Sample description

The study team visited 365 pregnant women from the original list of 400 who had been registered in CHW monitoring books; all consented to an observed CHW home visit during pregnancy. The subsequent follow-up interview three months later revealed that one woman had died, and two women had travelled away from the study area; 362 women were matched for analysis. Table 1 presents the characteristics of women who participated in the final study. The median age of participants was 25 years (interquartile range (IQR) 20–29 years). Most women (95%) were married, 51% had had at least four previous pregnancies, and 89% were above 4 months of gestation at the observed home visit. Just 18 women (5%) had only recently been registered in the record books and were receiving their first CHW home visit during the observation. Seventy-five per cent of women had already attended antenatal care at least once for the current pregnancy. Half of the women had some formal education, with the average duration of school attendance being 9 years.

### CHW counselling skills

The overall median home visit duration was 40 min (interquartile range 30–46). Table 2 summarizes the frequency of positive counselling skills observed during the accompanied home visits. Prevalence was generally very high with 10 of the positive counselling skills being observed at more than 90% of home visits. Fewer than half the CHW home visits included a review of patient history (33%), enlisting family support (41%), exploring for additional information (41%) and checking for pregnant women’s concerns (46%). The score to summarise counselling skills ranged from 9 to 18, mean score was 14.3 (standard deviation 2.6). In Table 3 we observe that the mean counselling score was higher for the least poor women compared to the poorest women (*p* = 0.04), for married compared to unmarried women (*p* = 0.03), and for women who had previously attended facility-based ANC that pregnancy compared to women who had not (*p* < 0.001).

### Validity of maternal recall for CHW content of care

The frequencies of all 60 content of care indicators are shown in the Additional file 1. Ten indicators had more than 5 values per cell in the 2 × 2 tables and therefore met the eligibility criteria for the validity analysis (Table 4). The number of don’t know responses was very low. Compared to observers, women reported higher frequencies for all 10 indicators. Consistent with this we observed high sensitivity > 70% but low specificity and low individual-level accuracy (AUC < 0.6) for all 10 indicators. The accuracy of just one indicator, whether the CHW checked the woman’s blood pressure during the home visit, was positively influenced by a higher counselling score (β = 0.021, 95% CI (0.001–0.041)), although CHW counselling quality explained only a very small proportion of the observed accuracy of maternal responses (R-squared = 0.0157).

## Discussion

Home visits by CHWs play an important role in promoting healthy behaviours for pregnancy, childbirth, and the postnatal period, and accurate maternal reports of the content of care are central to monitoring progress. Our study in Gombe State analyzed the validity of 10 CHW content of care indicators and found that, relative to observers, women consistently over-reported the frequency of indicators, resulting in an overall assessment of poor validity when asked to recall the content of a specific home visit. We were also interested to examine the effect of CHW counselling skills on recall but observed no relationship between high scores for counselling skills and better accuracy of maternal reporting.

CHWs had been trained to deliver up to 60 health education messages or health checks to the women they visited, depending on the stage of pregnancy of individual women. Notably, during observations, many of these had very high observed frequency; it is likely that these findings do not represent accurate estimates of coverage because of the Hawthorne effect incurred through observation [30]. The CHWs were likely demonstrating the best work that they were able to and this has interesting programmatic implications because it highlights the actions that were not carried out even under observation conditions.

We were keen to understand the counselling skills that CHWs demonstrated during home visits and had trained the observers to document the presence or absence of 18 positive skills. Reflecting on the findings we have broadly categorized these as being inter-personal or technical. Inter-personal counselling skills, for example making appropriate introductions, using appropriate language, making eye contact and smiling were very frequently observed. But the more technical counselling skills, for example, reviewing the woman's history, probing for additional information or checking for understanding were less frequent: these weaker areas present an opportunity to strengthen the CHW scheme. Of concern, we also found that CHW counselling quality differed between subgroups of women, with fewer positive skills demonstrated during visits to the poorest and the unmarried women in the sample. In the Gombe scheme CHWs are selected from their communities and are known to the pregnant women they visit; the quality of the relationship between the CHWs and the women could also influence the quality of CHW services to poor communities [31, 32]. Attitudinal barriers arising from the CHWs personal and societal beliefs could result in unfavourable attitudes towards some women in the community such as unmarried women, and direct action could be taken to try to address this.

Our study adds to the growing body of validity evidence around the accuracy of women’s reports about health services received during pregnancy, birth and the postnatal period, much of it suggesting that reports are often invalid, perhaps being most accurate for physical examinations, but being especially poor for events around the time of birth. Few studies have examined the accurate recall of health education messages (or counselling) but there is the suggestion that the wording of these questions might be subject to misinterpretation by women [33]. We explicitly focused on community-based, not facility-based care, and the recall of health education messages and health checks received by women in their own homes during pregnancy. In doing so we experienced limitations in applying criterion validation methods to examine community-based behaviours that lead us to make only cautious interpretations of our findings and these are discussed below.

Measurement of both the reference standard and the self-report measures may have been imperfect. For the reference standard that relied on observation, the Hawthorne effect is likely to have been present and we think may be particularly acute given the context of being in CHWs own communities where the presence of a study team might have a strong influence on perceived status. In this study the excellent demonstration of the content of care by many CHWs resulted in little heterogeneity of responses and some very small numbers in the two-by-two validation tables, limiting the number of variables available for the analysis.

We observed variability in the counselling skills demonstrated by CHWs by the characteristics of women visited: more detailed data on the characteristics of the CHWs would have been useful to examine whether this was a systematic response to the status of women or whether less capable CHWs happened to be operating in areas with relatively more vulnerable populations.

For the self-reported measures, there was the indication of a strong social desirability bias amongst women who consistently overreported the care they had received from CHWs who lived in their communities. In addition, for the self-report measures, we were asking women to relate the responses to a precise CHW home visit that we had observed in the past and it cannot be discounted that women were unsure about the exact reference point. Both these limitations would be likely to systematically inflate the self-reported frequencies leading to a larger number of false positives and masking the true validity. We cannot be sure whether women cannot give accurate responses about care received in their own homes or whether, in the context of this study, there were pressures not to give accurate responses. Finally, we were not able to identify a validated tool with which to assess the quality of counselling skills, although such a tool would be useful given the prominence given to the role of CHWs to communicate essential health messages to families.

## Conclusions

The CHWs observed in this study demonstrated strong inter-personal counselling skills but more technical counselling skills to check on client history or concerns were less frequently observed. Irrespective of counselling skills, we found that women consistently overestimated CHW content of care, indicating an overall assessment of poor validity. But methodological challenges lead us to be reflective about the transferability of criterion validity methods in the community. We recommend a need for caution when drawing conclusions regarding the validity of women’s survey responses for tracking community-based services, and the need to apply additional methods to track CHW activities.

## Supplementary Information

**Additional file 1.**

## Data Availability

The data are available from the principal investigator of the IDEAS project and co-author for this manuscript, Prof Tanya Marchant ORCID id 0000–0002-4228–4334. Reuse permitted on request.

## Abbreviations

ANC: Antenatal Care
AUC: Area Under Receiver Curve
CHW: Community Health Worker
DHS: Demographic Health Surveys
IDEAS: Informed Decisions for Actions to improve maternal and newborn health
LMIC: Low-Middle Income Country
LSHTM: London School of Hygiene & Tropical Medicine
MNH: Maternal and Newborn Health
MICS: Multiple Indicator Cluster Surveys
