# Supplementary material for: How valid are women’s reports of the antenatal health services they receive from Community Health Workers in Gombe State north-eastern Nigeria?
Source: BMC Pregnancy Childbirth. 2022 Dec 3;22:898. doi: 10.1186/s12884-022-05220-x (PMC9719641; doi:10.1186/s12884-022-05220-x)
Supplement: Supplementary file 1 — Additional file 1. [file 12884_2022_5220_MOESM1_ESM.docx]

**Validity analysis results for all 60 CHW content of care indicators during home visits to pregnant women in Gombe State, Nigeria**

| **Indicator** | **Observed coverage**  **N (%)** | **Women’s report**  **N (%)** | **Matched Pairs**  **N** | **Don’t Know**  **N** | **≥ 5 counts per cell** | **Agreement %**  **(95% CI)** | **Sensitivity**  **%**  **(95% CI)** | **Specificity**  **%**  **(95% CI)** | **AUC**  **(95% CI)** | **ROC regression coefficient***  **(β 95% CI)** |
| --- | --- | --- | --- | --- | --- | --- | --- | --- | --- | --- |
|  | **N=362** | |  |  |  |  |  |  |  |  |
| **CHW health education messages about ANC and pregnancy danger signs** | | | | | | | | | | |
| Importance of ANC attendance | 355 (98.0) | 361 (99.7) | 362 | 0 | N | 97.8  (96.3-99.3) | - | - | - | - |
| Early/immediate ANC attendance | 318 (87.8) | 362 (100) | 362 | 0 | N | 87.9  (84.5-91.2) | - | - | - | - |
| Encourage at least four ANC health facility visits | 290 (80.1) | 361 (99.7) | 362 | 0 | N | 80.3  (76.2-84.5) | - | - | - | - |
| **CHW health education messages on the purpose of ANC** | | | | | | | | | | |
| Counselling and education | 296 (81.7) | 361 (100) | 361 | 1 | N | 81.7  (77.7-85.6) | - | - | - | - |
| Assessing baby’s age and growth | 259 (71.5) | 361 (99.7) | 362 | 0 | N | 70.2  (65.4-74.9) | - | - | - | - |
| Monitor progress of pregnancy | 308 (85.0) | 361 (99.7) | 362 | 0 | N | 94.5  (92.1-96.8) | - | - | - | - |
| Manage any complications | 279 (77.0) | 362 (100) | 362 | 0 | N | 76.2  (71.8-80.7) | - | - | - | - |
| Check for problems (e.g., blood pressure & anaemia) | 347 (95.8) | 357 (98.6) | 362 | 0 | N | 94.5  (92.1-96.8) | - | - | - | - |
| Provide drugs and injections | 342 (94.4) | 349 (96.4) | 362 | 0 | N | 91.4  (88.5-94.3) | - | - | - | - |
| **CHW health education messages to promote good nutrition and sanitation in pregnancy** | | | | | | | | | | |
| CHW promote good nutrition in pregnancy | 358 (98.8) | 362 (100) | 362 | 0 | N | 98.9  (97.8-100) | - | - | - | - |
| CHW promote importance of good sanitation | 355 (98.0) | 362 (100) | 362 | 0 | N | 98.1  (96.6-99.5) | - | - | - | - |
| **CHW health education messages about danger signs in pregnancy** | | | | | | | | | | |
| Did CHW talk to pregnant woman about danger signs? | 358 (98.8) | 362 (100) | 362 | 0 | N | 98.9  (97.8-100) | - | - | - | - |
| **Which danger signs did she talk about?** | | | | | | | | | | |
| Swollen face and hands in pregnancy | 354 (97.8) | 360 (99.4) | 362 | 0 | N | 97.2  (95.5-98.9) | - | - | - | - |
| Abdominal and backache is pregnancy | 333 (91.9) | 361 (99.7) | 362 | 0 | N | 92.3  (89.5-95.0) | - | - | - | - |
| Fits and convulsions in pregnancy | 313 (86.5) | 360 (99.7) | 361 | 1 | N | 86.7  (81.2-90.3) | - | - | - | - |
| Bleeding in pregnancy | 356 (98.3) | 358 (99.1) | 361 | 1 | N | 97.2  (95.5-98.9) | - | - | - | - |
| Excessive backache in pregnancy | 343 (94.8) | 358 (98.9) | 362 | 0 | N | 93.7  (91.1-96.2) | - | - | - | - |
| Malaria and fever in pregnancy | 342 (94.4) | 358 (98.9) | 362 | 0 | N | 94.2  (91.8-96.6) | - | - | - | - |
| Swollen legs | 322 (88.9) | 358 (98.9) | 362 | 0 | N | 87.9  (84.5-91.3) | - | - | - | - |
| **CHW health education messages about birth preparedness and facility delivery** | | | | | | | | | | |
| CHW provided education on birth preparedness and facility delivery? | 358 (98.8) | 361 (99.7) | 362 | 0 | N | 98.6  (97.4-99.8) | - | - | - | - |
| **Which birth preparedness items did the CHW mention?** | | | | | | | | | | |
| Sanitary pad | 284 (78.4) | 359 (99.4) | 361 | 0 | N | 78.9  (74.7-83.2) | - | - | - | - |
| Soap | 316 (87.2) | 360 (99.4) | 362 | 0 | N | 87.9  (84.5-91.2) | - | - | - | - |
| Prepare skin disinfectant fluid | 103 (28.4) | 338 (93.8) | 360 | 2 | Y | 30.3  (25.5-35.1) | 92.2  (85.3-96.6) | 5.4  (3.0-9.0) | 0.49  (0.46-0.52) | -0.003  (-0.014-0.008) |
| Cord clamp or thread | 292 (80.6) | 358 (99.1) | 361 | 1 | N | 80.9  (76.8-84.9) | - | - | - | - |
| Hand gloves | 321 (88.6) | 357 (99.2) | 360 | 2 | N | 88.3  (84.9-97.1) | - | - | - | - |
| Towels or clothes | 334 (92.2) | 357 (98.8) | 361 | 1 | N | 91.1  (88.2-94.1) | - | - | - | - |
| Saving cash for pay of expenses | 339 (93.6) | 359 (99.7) | 360 | 2 | N | 95.6  (93.4-97.8) | - | - | - | - |
| Arranging transport to facility | 214 (59.1) | 358 (99.1) | 361 | 1 | N | 58.5  (53.5-63.7) | - | - | - | - |
| Education/information on emergency transport scheme | 339 (93.6) | 349 (96.4) | 362 | 0 | N | 90.1  (86.9-93.2) | - | - | - | - |
| Provided emergency telephone number | 173 (47.7) | 258 (72.4) | 356 | 6 | Y | 50.0  (44.7-55.3) | 73.3  (66.0-79.7) | 28.3  (21.9-35.4) | 0.51  (0.46-0.55) | 0.006  (-0.019-0.031) |
| Did the CHW promote facility delivery? | 354 (97.7) | 361 (99.7) | 362 | 0 | N | 97.5  (95.9-99.1) | - | - | - | - |
| **CHW health education messages about care for the newborn** | | | | | | | | | | |
| Did the CHW provide education on essential newborn care? | 361 (99.7) | 358 (99.4) | 360 | 2 | N | 99.2  (97.9-100) | - | - | - | - |
| **CHW health education messages about care for the newborn** | | | | | | | | | | |
| The baby needs to be kept warm | 341 (94.1) | 359 (99.1) | 362 | 0 | N | 93.4  (90.8-95.9) | - | - | - | - |
| The baby should not be bathed in the first 24 hours | 354 (97.8) | 361 (99.7) | 362 | 0 | N | 97.5  (95.9-99.1) | - | - | - | - |
| The baby needs vaccination | 302 (83.4) | 362 (100) | 362 | 0 | N | 83.4  (79.6-87.3) | - | - | - | - |
| Nothing should be put on the baby’s cord except (chlorhexidine) gel | 340 (93.9) | 357 (98.8) | 361 | 1 | N | 92.8  (90.1-95.5) | - | - | - | - |
| The baby needs to be fed | 294 (81.2) | 360 (99.7) | 361 | 1 | N | 80.9  (76.8-84.9) | - | - | - | - |
| Breastfeeding should start immediately/ within 30 minutes after birth | 307 (84.8) | 358 (98.8) | 362 | 0 | N | 84.3  (80.5-88.0) | - | - | - | - |
| The baby’s first breastfeed should be the colostrum (mother’s first milk) | 315 (87.1) | 361 (99.7) | 362 | 0 | N | 86.7  (83.2-90.3) | - | - | - | - |
| Baby should not be fed any other feeds; no water, no local foods | 325 (89.8) | 361 (100) | 361 | 1 | N | 89.8  (86.6-92.9) | - | - | - | - |
| The baby should be exclusively breastfed for 6 months | 338 (93.4) | 361 (99.7) | 362 | 0 | N | 93.1  (90.5-95.7) | - | - | - | - |
| Did the CHW provide education on newborn danger signs? | 322 (88.9) | 354 (98.6) | 359 | 3 | N | 88.9  (85.5-92.2) | - | - | - | - |
| **CHW health education messages about newborn danger signs and illness** | | | | | | | | | | |
| The baby moves only when touched/is too sleepy | 272 (75.1) | 342 (95.5) | 358 | 4 | N | 77.7  (73.2-82.1) | - | - | - |  |
| The baby does not feed well | 293 (80.9) | 346 (96.9) | 357 | 5 | N | 82.1  (77.9-86.2) | - | - | - | - |
| Baby is feverish | 310 (85.6) | 351 (97.5) | 360 | 2 | N | 86.4  (82.8-80.0) | - | - | - | - |
| Baby has difficulty breathing | 298 (82.3) | 351 (97.2) | 361 | 1 | N | 83.7  (79.8-87.5) | - | - | - | - |
| Baby’s cord is red | 280 (77.3) | 349 (96.6) | 361 | 1 | Y | 77.0  (72.6-81.4) | 97.5% (94.9%-99.0%) | 6.2%  (2.0%-13.8%) | 0.52  (0.49-0.55) | 0.02  (-0.003-0.044) |
| Pneumonia as a serious illness of the lungs | 296 (81.7) | 345 (96.6) | 357 | 5 | Y | 82.4  (78.3-86.5) | 97.6% (95.2%-99.0%) | 11.1%  (4.6%-21.6%) | 0.54  (0.50-0.58) | -0.002  (-0.035-0.032 |
| Diarrhoea when baby passes loose or watery stools | 293 (80.9) | 345 (96.1) | 359 | 3 | N | 83.0  (79.0-86.9) | - | - | - | - |
| Malaria as a serious infection that can kill a child | 305 (84.3) | 356 (98.3) | 362 | 0 | N | 84.3  (80.5-88.0) | - | - | - | - |
| Seeking care from a health facility for sick newborns | 318 (87.8) | 357 (98.6) | 362 | 0 | N | 87.0  (83.5-90.5) | - | - | - | - |
| Provide information on the importance of immunisation | 312 (86.1) | 359 (99.4) | 361 | 1 | N | 85.6  (81.9-89.3) | - | - | - | - |
| **CHW health education messages to about maternal illnesses** | | | | | | | | | | |
| HIV infection | 296 (81.7) | 343 (95.0) | 361 | 1 | Y | 82.3  (78.6-86.5) | 97.3% (94.7%-98.8%) | 15.4%  (7.6%-26.5%) | 0.56 (0.52-0.61) | -0.019  (-0.058-0.02) |
| Diabetes | 268 (74.0) | 335 (93.8) | 357 | 5 | Y | 75.3  (70.8-79.9) | 96.3% (93.2%-98.2%) | 13.3%  (7.1%-22.1%) | 0.55 (0.51-0.59) | 0.02  (-0.011-0.052) |
| Hypertension | 296 (81.7) | 345 (95.3) | 362 | 0 | Y | 80.4  (76.3-84.5) | 96.3% (93.4%-98.1%) | 9.1%  (3.4% -18.7%) | 0.53 (0.49-0.56) | 0.014  (-0.018-0.047) |
| **CHW health checks or therapeutic care carried out during the home visit** | | | | | | | | | | |
| Dispense folic acid and/or iron | 29 (8.0) | 260 (71.8) | 362 | 0 | N | 33.9  (29.1-38.9) | - | - | - | - |
| Dispense misoprostol | 25 (6.9) | 224 (61.8) | 362 | 0 | Y | 42.2  (36.1-46.3) | 72.0% (50.6%-87.9%) | 38.9% (33.6%-44.3%) | 0.55 (0.46-0.65) | -0.016  (-0.037-0.004) |
| Dispense chlorhexidine | 30 (8.2) | 225 (62.3) | 361 | 1 | Y | 41.4  (36.3-46.5) | 73.3%  (54.1%-87.7%) | 38.7% (33.4%-44.2%) | 0.56  (0.48-0.64) | -0.011  (-0.032-0.009) |
| Measure woman’s temperature | 10 (2.7) | 260 (72.0) | 361 | 1 | N | 28.5  (23.9-33.2) | - | - | - | - |
| Measure woman’s blood pressure | 81 (22.3) | 283 (78.3) | 361 | 1 | Y | 37.9  (32.9-42.9) | 86.4% (77.0%-93.0%) | 23.9%  (19.1%-29.4%) | 0.55 (0.51-0.60) | 0.021  (0.001-0.041) |
